# Seminal vesicle metastasis after partial hepatectomy for hepatocellular carcinoma

**DOI:** 10.1186/1471-2407-11-111

**Published:** 2011-03-28

**Authors:** Li Gong, Minwen Zheng, Yanhong Li, Wendong Zhang, Wangjun Bu, Lifang Shi, Wei Zhang, Hong Yan

**Affiliations:** 1 The Helmholtz Sino-German Laboratory for Cancer Research, Department of Pathology, Tangdu Hospital, the Fourth Military Medical University, 710038 Xi'an, Shaanxi Province, PR China; 2 Department of Radiology, Xijing Hospital, the Fourth Military Medical University, 710032 Xi'an, Shaanxi Province, PR China; 3 Department of General Surgery, the Second Affiliated Hospital, Xi'an Jiaotong University, 710031 Xi'an, Shaanxi Province, PR China; 4 Department of Radiology, the Second Affiliated Hospital, Xi'an Jiaotong University, 710031 Xi'an, Shaanxi Province, PR China; 5 Depatment of Ophthalmology, Tangdu Hospital, the Fourth Military Medical University, 710038 Xi'an, Shaanxi Province, PR China

**Keywords:** seminal vesicle, hepatocellular carcinoma, metastasis, clinical pathology

## Abstract

**Background:**

Metastasis to the seminal vesicle is extremely rare for hepatocellular carcinoma (HCC). To our knowledge, it has been not reported in literature. The purpose of the present paper was to report a case of metastasis to the seminal vesicle after HCC resection, along with its histological features and immunohistochemical characteristics.

**Case Presentation:**

A 46-year-old Chinese man was admitted to our hospital due to abdominal distension. He had a history of HCC related to hepatitis B virus infection. Moreover, left partial hepatectomy was performed in another hospital 28 months ago, and right partial hepatectomy for HCC recurrence in our hospital 4 months ago. After resection, radiofrequency ablation therapy had been performed. About 27 months after the initial operation, contrast-enhanced computed tomography (CT) of the pelvic cavity revealed a mass with homogeneous enhancement in the seminal vesicle. Transrectal needle biopsy revealed a poorly differentiated adenocarcinoma. Therefore, seminal vesiculectomy was resected. The histological diagnosis of the removed tumor was compatible with the original HCC. Immunohistochemical examination demonstrated that the tumor cells were positive for glypican-3 (GPC3), alpha-fetoprotein (AFP), hepatocyte paraffin-1 (Hep Par 1), cytokeratin 18 (CK 18), and hepatocyte antigen, which confirmed that the seminal vesicle tumor was a metastatic tumor of HCC. However, CT subsequently revealed multiple metastatic foci in the abdominal and pelvic cavities in May 2009 and August 2009, respectively.

**Conclusion:**

The seminal vesicle is an extremely rare metastatic site for HCC, and the prognosis is very poor. A combination of clinical and pathological features is necessary for a correct diagnosis, and primary tumor should be excluded before diagnosing metastatic foci.

## Background

Hepatocellular carcinoma (HCC) is one of the most common malignancies worldwide, accounting for nearly one million new cases each year [1]. The long-term prognosis for HCC remains poor, with a 5-year survival rate of < 5% [2], and intrahepatic and extrahepatic metastasis are the most important factors. The liver is the most common site of HCC metastasis, accounting for approximately 85% to 90% of all cases [3]. Extrahepatic metastases have been reported to occur in 13.5% to 42% of HCC patients [4-6]. The most frequent sites of extrahepatic metastases are lung, abdominal lymph node, and bone [4,7,8]. To our knowledge, it has not been reported in literature that HCC metastasize to the seminal vesicle. Here we report the case of a 46-year-old man with metastatic HCC to the seminal vesicle after a liver resection for HCC and recurrence, and observed its histological and immunohistochemical characteristics.

## Case presentation

**Figure 1:**
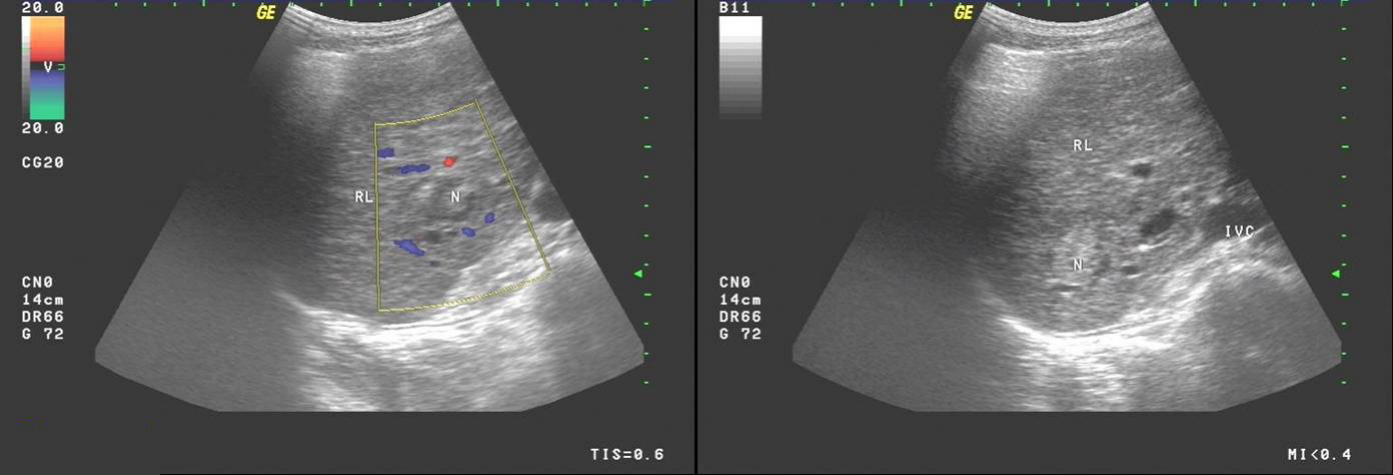
**US image shows that the echogenic dots thicken slightly, and the echo strengthen slightly**. A well-defined and regular-appearance echo zone (2.7 cm × 2.3 cm) is found in the right lobe of the liver. Moreover, A well-defined, and heterogeneous hyperechoic mass (2.1 cm × 2.0 cm) is found in the right lobe neighboring to the porta hepatis.

**Figure 2:**
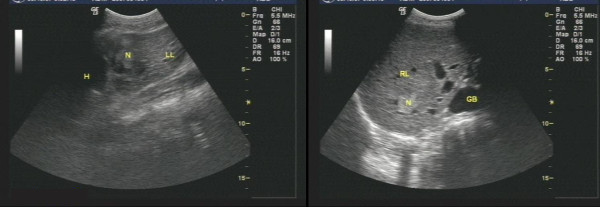
**US image shows that the echogenic dots are intensive, dim, and mal-distributed**. An ill-defined and irregular dense echo zone (3.7 cm × 3.3 cm) is found in left lobe, and the echogenic dots are confused and disorderly. Moreover, a dense echo zone (1.8 cm × 1.7 cm) is found in right posterior lobe, and the internal echo are well-distributed.

**Figure 3:**
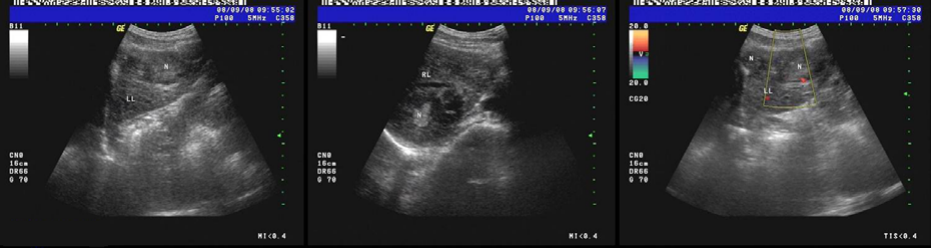
**US image shows that the echogenic dots are intensive, dim, and heterogeneous**. Multiple dense echo zones with inequality of size are found, and the larger (5.0 cm × 3.1 cm) locats in the left lobe, and the internal echo is well-distributed insufficiently.

**Figure 4:**
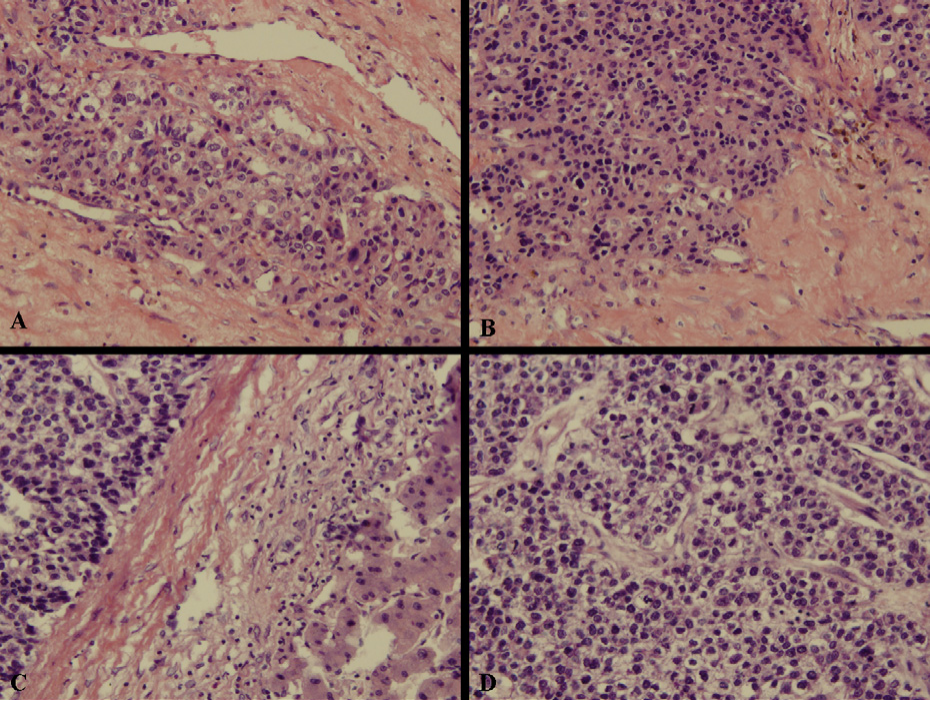
**(A, B) A microscopic view of the tumor in the seminal vesicle: the tumor cells arranged in trabecular and solid, which is compatible with the original HCC (C, D)**.

**Figure 5:**
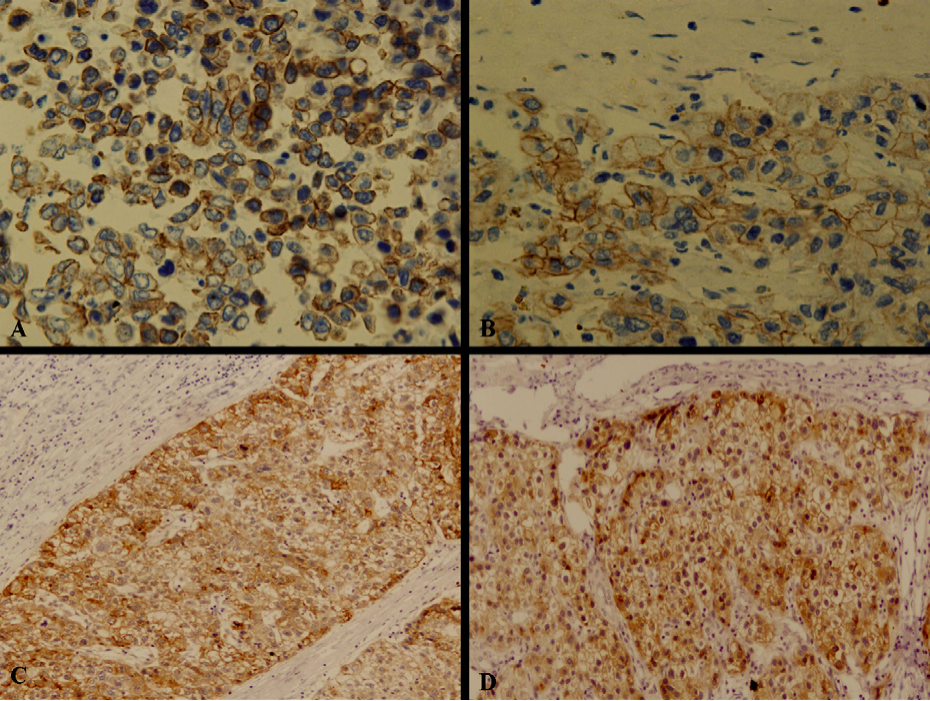
**The tumor cells from the seminal vesicle (A, B) and liver (C, D) were positive for GPC3**.

**Figure 6:**
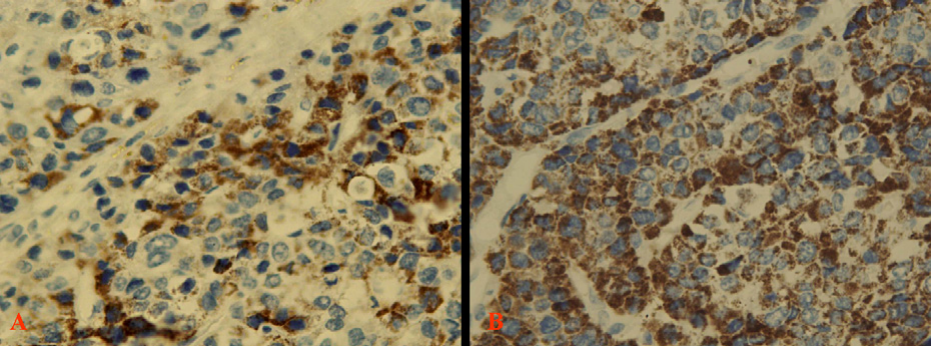
**The tumor cells from the seminal vesicle (A) and liver (B) were positive for AFP**.

**Figure 7:**
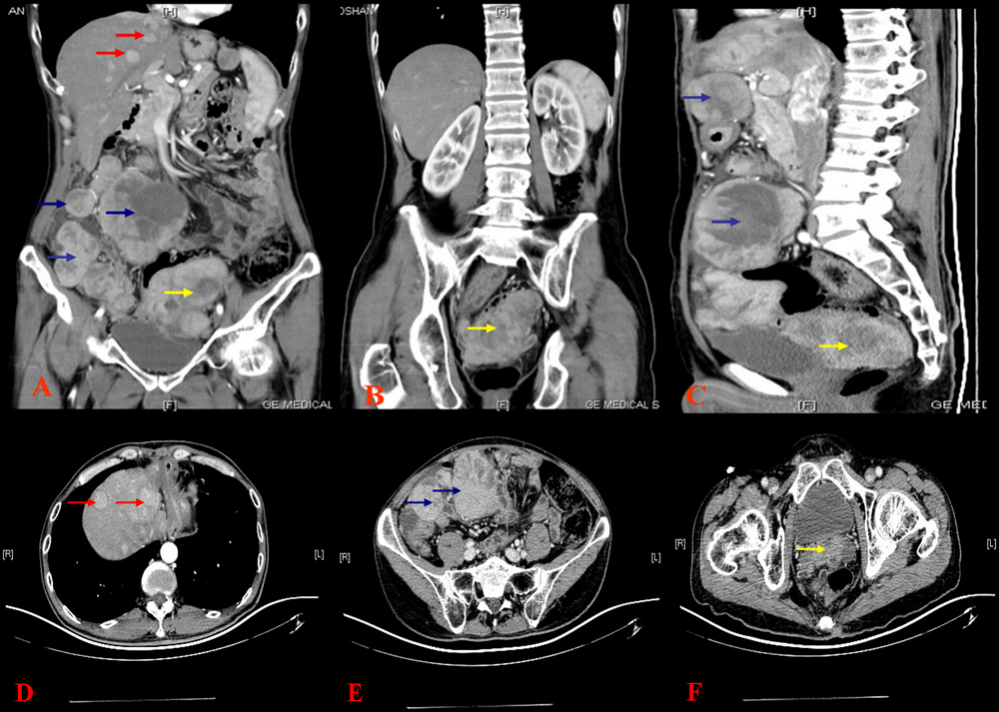
**Computed tomography (CT) revealed multiple intrahepatic recurrent or metastatic foci and metastatic foci in abdominal and pelvic cavity**. Red arrow, intrahepatic recurrent or metastatic foci; blue arrow, metastatic foci in abdominal; yellow arrow; metastatic foci in the seminal vesicle region.

A 46-year-old Chinese man was admitted to our hospital due to abdominal distension in January 2009. It was the second time that he was as an inpatient of our hospital. The patient had a history of hepatitis B for approximately ten years. Moreover, his mother, two older brothers, and older sister had histories of hepatitis B. Among them, one older brother died from HCC three years ago. He began to notice his weight loss since June 2006, and performed a health examination in local hospital in August 2006. Laboratory test results included the following: white blood cell count, 4800/μl; hemoglobin, 10.3 g/dl; platelets, 84 × 10^3^/μl; aspartate aminotransferase, 42 U/L; alanine aminotransferase, 30 U/L (Table 1). Hepatitis B surface antigen, hepatitis B e antigen, and hepatitis B core antibody were positive. The level of serum alpha-fetoprotein (AFP) was elevated to 3350 ng/ml (normal level < 20 ng/ml), whereas serum carcinoembryonic antigen (CEA) and human chorionic gonadotropin (HCG) levels were within normal limits. Abdominal ultrasound examination revealed a solitary mass, which was determined to be HCC by liver needle biopsy. Sequentially, the patient underwent partial resection of the left anterior segment of the liver (about 2 cm in diameter) in local hospital in September 2006. After leaving the hospital, the patient received thymic peptide injections twice a week, but the dosage was not provided. About half a year after the initial operation, abdominal ultrasound examination revealed a mass in the right lobe of the liver, which was determined to be recurrent HCC (Figure 1). Thus, regular (one time/three months) radiofrequency ablation therapy (60W, 2 min/time; the total 3 times) and transarterial interventions (embolization or chemoembolization) were performed until September 2008. During therapy, the serum level of AFP was elevated (21.51-4689 ng/ml) (the details could be seen in table 1). However, abdominal ultrasound examination also revealed multiple solid occupation in the liver (Figure 2 and 3). Thus, the patient underwent partial resection of the right anterior segment for HCC recurrence in our hospital on September 20, 2008. After operation, the patient was not received any antitumoral therapy due to his weakness. On December 23, 2008, he went to see a doctor for abdominal distension in local hospital. Contrast-enhanced computed tomography (CT) of the pelvic cavity revealed a mass (5.0 cm in diameter) with homogeneous enhancement in the seminal vesicle. Transrectal needle biopsy revealed a poorly differentiated adenocarcinoma with histological characteristic similar to that of the original HCC. Considering the patient's history, the doctors diagnosed the mass in the seminal vesicle as a metastatic focus. Unfortunately, the CT image and biopsy were not obtained from the other hospital. In order to receive further treatment, he came to our hospital. During the operation, only a mass with the size of 4.8 cm in diameter was found in left seminal vesicle, but involved right seminal vesicle. Thus, bilateral seminal vesicle and prostate were resected. Macroscopically, the structure of the seminal vesicle was unclear and replaced by tumor tissues with hemorrhage. Histologically, the removed tumor was found to be compatible with the original HCC. Tumor cells arranged in trabecular, solid, or tubular patterns. Nuclei and nucleoli were prominent, and the cytoplasm was scanty and basophilic (Figure 4). Moreover, immunohistochemical examination demonstrated that the tumor cells were positive for glypican-3 (GPC3) (Figure 5), AFP (Figure 6), hepatocyte paraffin-1 (Hep Par 1), cytokeratin (CK)18, and hepatocyte antigen, but negative for CK7, CK20, placental alkaline phosphatase (PLAP), prostate-specific antigen (PSA), cancer antigen 125 (CA125), epithelial membrane antigen (EMA), cluster of differentiation (CD) 117, and CEA, which confirmed that the seminal vesicle tumor was metastatic HCC. In May 2009, a abdominal CT reviewing revealed multiple metastatic foci in the abdominal cavity in local hospital. Thus, the patient came to our hospital again in order to receive further therapy by the iatrotechnique of microwave. Two solid and bad mobility masses, with the size of 6 cm, were touched in the lower abdomen by physical examination. Laboratory test results included the following: white blood cell count, 4890/μl; red blood cell count, 4490 × 10^3^/μl; hemoglobin, 13.3 g/dl; platelets, 174 × 10^3^/μl; aspartate aminotransferase, 25 U/L; alanine aminotransferase, 19 U/L. The level of serum AFP was elevated to 4689 ng/ml (Table 1). Abdominal ultrasound examination revealed that there was a dense echo zone in right anterior lobe and right posterior lobe of the liver, respectively. Moreover, a low echo area with clear circumscription was found at the ahead of head of pancreas. Thus, all these foci were treated by microwaves. However, CT imaging revealed multiple metastatic foci in the abdominal and pelvic cavities in September 2009 (Figure 7). Of course, the Written informed consent was obtained from the patient for publication of this case report and any accompanying images.

**Table 1:** Laboratory test results of blood routine examination, liver function and serous AFP at different time

Date	**WBC(10**^**9**^**/l)**	**RBC(10**^**12**^**/l)**	HGB(g/l)	**PLT(10**^**9**^**/l)**	ALT(u/l)	AST(u/l)	AFP(ng/ml)
06.09	4.80	3.85	103	84	30	42	3350
07.03	3.62	4.32	121	88	28	40	2249
07.09	5.03	4.55	139	82	32	38	897
08.09	4.71	4.40	135	135	135	78	772
09.01	4.43	3.44	103	125	14	20	3350

09.08	4.89	4.49	133	174	19	25	4689

## Discussion

Metastasis from the liver to extrahepatic tissues is not common for HCC (13.5%-42% of cases) and has a poor prognosis [4-6]. For unknown reasons, the seminal vesicle is an extremely rare site of metastasis for HCC; therefore, clinical data is limited.

Tumors of the seminal vesicles may be primary tumors or secondary tumors originating from adjacent organs such as the bladder, prostate, or rectum. Primary seminal vesicle tumors are rare and may be benign (papillary adenoma, cystadenoma, hydatid cyst, and amyloid deposition) or malignant (adenocarcinoma, sarcoma, cystosarcoma phyllodes, primary seminoma, and carcinoid). Adenocarcinoma is the most frequent malignant tumor. Therefore, we first excluded it before diagnosing the mass as metastasis from HCC. In 1956, Dalgaard and Giertson [9] established the following criteria for the diagnosis of primary seminal vesicle adenocarcinoma: 1) the tumor should be a microscopically verified carcinoma, localized exclusively or mainly to the seminal vesicle; 2) the presence of other simultaneous primary carcinoma should be excluded; and 3) the tumor should preferably resemble the architecture of the non-neoplastic seminal vesicle.

In the present case, the patient had a history of HCC. Moreover, according to the surgical doctor's introduction, the mass was only found in seminal vesicle during the operation. In addition, the histopathological characteristics of the mass from seminal vesicle were similar to that of primary HCC, although it had a poorly differentiated appearance. Immunohistochemically, the tumor cells were positive for GPC3, AFP, hepatocyte antigen, HepPar1, and CK18, but negative for CK7, CK20, PLAP, PSA, CA125, EMA, CD117, and CEA. Primary seminal vesicle adenocarcinoma expresses these markers as well as CK7, CA125 and CEA but not AFP, GPC3 and hepatocyte antigen [10]. It is known to all, AFP is a relatively specific but rather insensitive marker for hepatocellular differentiation and is present in only one quarter of the cases. Recently, GPC3 has been reported as novel serum and histochemical marker for HCC, with positive staining in 72% to 100% of cases [11-16]. Thus, based on the tumor cells positive reactivity for GPC3, AFP, hepatocyte antigen, Hep Par 1, and CK18, we thought that the seminal vesicle mass should firstly be considered as a metastatic HCC lesion. Of course, AFP, HepPar1 and GPC3 were not only positive in HCC. Many malignant tumors with AFP producing and hepatoid features resembling HCC, such as hepatoid adenocarcinoma of the stomach and primary hepatoid yolk sac tumor (H-YST) of ovary and testis, especially the latter, express AFP, Hep Par 1 and GPC3 [17,18]. Namely, AFP, Hep Par 1 and GPC3 are useful markers for HCC, but not entirely specific. However, the YST usually occurs in younger patients, and most of them occur in the ovaries and testis. About 20% arise in extraovarian sites, including the mediastinum, sacrococcygeal region, cervix, vulva, pelvis, and retroperitoneum [19-24]. We do not believe primary H-YST of the seminal vesicle has been reported in the literature. Moreover, gonadal dysgenesis and the presence of a residual component of typical yolk sac tumor or a polyvesicular vitelline pattern, or glandular-like structure some with mucin production, is helpful for differential diagnosis. In addition, immunohistochemically, the tumor cells of H-YSTs are positive for CK besides AFP and HepPar1, and negative for hepatocyte antigen, CK20, CEA and EMA. Thus, we finally considered it was a metastatic HCC lesion based on its clinical and pathological features.

No sufficient data is currently available regarding treatment for HCC with metastasis to the seminal vesicle. However, the prognosis appears to be poor. In our case, the patient had undergone regular radiofrequency ablation since the diagnosis of HCC, but intrahepatic and seminal vesicle metastatic lesions were found after half a year and 27 months, respectively. It was worse that CT revealed multiple metastatic foci in the abdominal and pelvic cavities after 32 months and 3 years, respectively.

In conclusion, the seminal vesicle is an extremely rare metastatic site for HCC, and the prognosis is very poor. Although AFP, Hep Par 1, hepatocyte antigen and GPC3 are useful markers for HCC. They are not, however, entirely specific, showing different extent staining in hepatoid adenocarcinoma and H-YST. Thus, a combination of clinical and pathological features is necessary for a correct diagnosis, and primary tumor should be excluded before diagnosing metastatic foci,

## Abbreviations

AFP: alpha fetoprotein; CA125: cancer antigen; CEA: carcinoembryonic antigen; CT: computed tomography; CK: cytokeratin; EMA: epithelial membrane antigen; GPC3: glypican 3; HBV: hepatitis B virus; HCC: hepatocellular carcinoma; HCV: hepatitis C virus; Hep Par 1: hepatocyte paraffin-1; PLAP: placental alkaline phosphatase, PSA: prostate-specific antigen;

## Competing interests

The authors declare that they have no competing interests.

## Authors' contributions

GL carried out the whole study and drafted the manuscript. ZMW, LYH, ZWD and HY participated in drafting the manuscript. BWJ provided the patient's history and performed the immunohistochemistry. ZMW and SLF collected the CT picture. ZW participated in its design and coordination and helped to draft the manuscript. All authors read and approved the final manuscript.

## Pre-publication history

The pre-publication history for this paper can be accessed here:

http://www.biomedcentral.com/1471-2407/11/111/prepub
